# Pharmacologic Ascorbic Acid as Early Therapy for Hospitalized Patients with COVID-19: A Randomized Clinical Trial

**DOI:** 10.3390/life12030453

**Published:** 2022-03-19

**Authors:** Dagan Coppock, Pierre-Christian Violet, Gustavo Vasquez, Katherine Belden, Michael Foster, Bret Mullin, Devon Magee, Isabelle Mikell, Lokesh Shah, Victoria Powers, Brian Curcio, Daniel Monti, Mark Levine

**Affiliations:** 1 Division of Infectious Diseases, Department of Medicine, Sidney Kimmel Medical College, Thomas Jefferson University, 1015 Chestnut Street, Philadelphia, PA 19107, USA; gvasquezmd@gmail.com (G.V.); katherine.belden@jefferson.edu (K.B.); 2 Molecular and Clinical Nutrition Section, National Institute of Diabetes and Digestive and Kidney Diseases, National Institutes of Health, 10 Center Drive, Bethesda, MD 20892, USA; pierre-christian.violet@nih.gov; 3 Jefferson Clinical Research Institute, Thomas Jefferson University, 833 Chestnut Street, Philadelphia, PA 19107, USA; mfoster610@gmail.com (M.F.); bret.mullin@jefferson.edu (B.M.); devon.magee@jefferson.edu (D.M.); 4 Sidney Kimmel Medical College, Thomas Jefferson University, 1015 Walnut Street, Philadelphia, PA 19107, USA; isabelle.mikell@students.jefferson.edu (I.M.); lokesh.shah@students.jefferson.edu (L.S.); tori.powers@students.jefferson.edu (V.P.); 5 Division of Biostatistics, Department of Pharmacology and Experimental Therapeutics, Sidney Kimmel Medical College, Thomas Jefferson University, 1015 Chestnut Street, Philadelphia, PA 19107, USA; curciob@arcadia.edu; 6 Department of Integrative Medicine and Nutritional Sciences, Sidney Kimmel Medical College, Thomas Jefferson University, 925 Chestnut Street, Philadelphia, PA 19107, USA; daniel.monti@jefferson.edu

**Keywords:** SARS-CoV-2, COVID-19, vitamin C, ascorbic acid

## Abstract

Despite the widespread availability of effective vaccines, new cases of infection with severe acute respiratory syndrome coronavirus-2, the cause of coronavirus disease 2019 (COVID-19), remain a concern in the settings of vaccine hesitancy and vaccine breakthrough. In this randomized, controlled, phase 2 trial, we hypothesized that high-dose ascorbic acid delivered intravenously to achieve pharmacologic concentrations may target the high viral phase of COVID-19 and thus improve early clinical outcomes. Sixty-six patients admitted with COVID-19 and requiring supplemental oxygen were randomized to receive either escalating doses of intravenous ascorbic acid plus standard of care or standard of care alone. The demographic and clinical characteristics were well-balanced between the two study arms. The primary outcome evaluated in this study was clinical improvement at 72 h after randomization. While the primary outcome was not achieved, point estimates for the composite outcome and its individual components of decreased use of supplemental oxygen, decreased use of bronchodilators, and the time to discharge were all favorable for the treatment arm. Possible favorable effects of ascorbic acid were most apparent during the first 72 h of hospitalization, although these effects disappeared over the course of the entire hospitalization. Future larger trials of intravenous ascorbic acid should be based on our current understanding of COVID-19 with a focus on the potential early benefits of ascorbic in hospitalized patients.

## 1. Introduction

In the United States, infection with severe acute respiratory syndrome coronavirus 2 (SARS-CoV-2) has led to an estimated 6.2 million hospitalizations and 767,000 deaths due to coronavirus disease 2019 (COVID-19) [1]. Despite the widespread availability of effective messenger ribonucleic acid vaccines, new infections remain a concern in the settings of vaccine hesitancy and vaccine breakthrough [2,3]. Furthermore, as new variants of SARS-CoV-2 emerge, such as the Delta variant, there is the risk of infection despite successful vaccination [4,5]. In these contexts, the development of new treatments for COVID-19 remains a priority.

COVID-19 pneumonia appears to be a two-phase disease, in which the first phase is dominated by a high viral load in the airways, followed by a later phase of low or undetectable virus and progressive organizing fibrosis [6]. We hypothesized that high-dose ascorbic acid (AA) delivered intravenously to achieve pharmacologic concentrations may target that early high viral phase through two distinct potential mechanisms: Lymphocyte activation and/or inhibition of early viral replication [7,8,9,10,11,12]. Both mechanisms are linked to hydrogen peroxide formation, for which pharmacologic AA concentrations serve as a prodrug [13,14,15,16]. When given intravenously, but not orally, pharmacologic AA concentrations increase the steady-state formation of hydrogen peroxide in vivo over hours in extracellular fluid but not in the blood [14,15].

To translate potential pharmacologic AA efficacy to acute COVID-19 treatment, we chose primary and secondary clinical endpoints that reflected these postulated mechanisms of action. These endpoints included clinical improvement within 72 h of pharmacologic AA treatment, including hospital discharge, the need for oxygen, the use of bronchodilators, days of fever, and clinical decline or serious adverse events. Here we present an exploratory, single-center 2:1 randomized open-label clinical study evaluating these endpoints in 66 people with acute COVID-19 admitted to one hospital system in the United States.

## 2. Methods

### 2.1. Study Design and Patients

This study was approved by the Thomas Jefferson University Office of Human Research Institutional Review Board (Protocol #20D.431). It was conducted in accordance with Federal-Wide Assurance #00002109 to the U.S. Department of Health and Human Services. Written informed consent was obtained from all patients. The study was registered at ClinicalTrials.gov (Identifier: NCT04363216). This prospective, randomized, open-label, phase II clinical trial was conducted at Thomas Jefferson University Hospital and Jefferson Methodist Hospital from 8 May 2020 through 31 December 2020. Potential study participants were identified based upon hospital admission with a new diagnosis of SARS-CoV-2. Subjects were screened by study personnel as soon after admission as feasible. Subjects were considered eligible for inclusion if they were male or a non-pregnant female, were greater than 18 years of age, were confirmed positive for SARS-CoV-2 infection by nasal swab, and required supplemental oxygen without an anticipated need for mechanical ventilation within 24 h. Patients were excluded if they had an estimated glomerular filtration rate less than 50 at the time of screening, known glucose-6-phosphate dehydrogenase deficiency, an anticipated need for mechanical ventilation within 24 h, a home oxygen requirement, known allergy to AA, or if they were enrolled in another COVID-19 treatment study at the time of enrollment. Patients who met screening requirements were approached for the study after discussion with their primary inpatient team (see Table S1 for CONSORT checklist).

### 2.2. Randomization

Once written consent was obtained, patients were randomized, using the REDCap Randomization Module, in a 2:1 ratio into either a treatment arm that received standard of care (SoC) plus AA or a control arm that received SoC alone. Randomization was stratified by risk of complications (high vs. low). Patients were considered high risk if they were older than 60 or had any of the following comorbidities: Hypertension, structural lung disease, cardiovascular disease, diabetes, immunocompromising conditions, or conditions requiring immunosuppressing medications.

### 2.3. Interventions

If randomized to the treatment arm, patients received routine clinical care plus up to 6 escalating doses of daily AA infusions in magnesium chloride to reduce the sensation of burning on injection. On day zero (the randomization day), patients received AA 0.3 g/kg intravenously once via a continuous 2-h infusion. On day one, patients received AA 0.6 g/kg intravenously once via continuous infusion. Thereafter, patients received 0.9 g/kg intravenously once daily through day five. Patients received these infusions while they were admitted. If patients were discharged prior to the final (day five) infusion, AA was discontinued at the time of discharge. If randomized to the control arm, patients received routine clinical care.

### 2.4. Procedures

While enrolled in the study, patients were continuously monitored by the study coordinator and investigators for adverse and serious adverse events. Patients were withdrawn if they progressed to a requirement of high-flow oxygen, were transferred to the intensive care unit, were intubated prior to completion of the study procedures, or if they developed any clinical condition that raised concerns for safety. An interim safety analysis was performed after the first 21 patients who were randomized to the treatment arm completed the course of AA.

### 2.5. Outcome Measures

The primary endpoint was a composite outcome—clinical improvement at 72 h post-randomization, defined as a 50% reduction in the highest flow rate of oxygen during the 72-h period, a 50% reduction in the most frequent use of bronchodilators within a 12-h window within the 72-h period, or hospital discharge (whichever came first).

The secondary endpoints analyzed included clinical decline within 36 h of randomization, defined as requiring ICU-level care; the rate of oxygen supplementation given, defined by the days to a 50% reduction in supplementation during the hospital course; any day of fever as defined by a temperature of greater than 100.4 °F; the time to discharge; and serious adverse events specific to the treatment. Adverse and serious adverse events were defined based upon guidelines set forth on clinicaltrials.gov [17].

### 2.6. Statistical Analyses

All analyses were prespecified in the trial protocol and followed the intention-to-treat principle, i.e., included all subjects as randomized. The protocol specified interim analyses based on the proportion of clinical decline among the first 21 patients in the AA group. The stopping rule specified that the trial would continue only if fewer than 5 of the first 21 patients experienced clinical decline requiring high-flow oxygen and/or ICU-level care; this corresponds to the one-sided upper 95% exact binomial confidence limit excluding the unacceptably high rate of 40%.

The main endpoint (clinical improvement within 72 h from randomization) was analyzed with a stratified Mantel–Haenszel procedure, with the risk of complication as the stratification factor. The comparison of the two trial groups was based on a 1-sided exact test with alpha 0.05; the corresponding 90% exact confidence interval was also computed. The dichotomous secondary endpoints of any fever and adverse events were analyzed similarly. The time to a 50% decrease in oxygen supplementation and time to discharge were analyzed with the Kaplan–Meier method and Cox proportional hazards regression. A secondary analysis of the time to discharge was performed, also on an intention-to-treat basis, with adjustments made for admission-to-randomization time.

### 2.7. Sample Size and Power

With a target sample size of 66 patients (44 in the AA group and 22 in the SoC group), the trial had 82% power to detect a difference of 60% vs. 25% clinical improvement in the AA vs. the SoC arm, using the one-sided Fisher’s exact test with alpha 0.05.

## 3. Results

### 3.1. Patients

**Figure 1:**
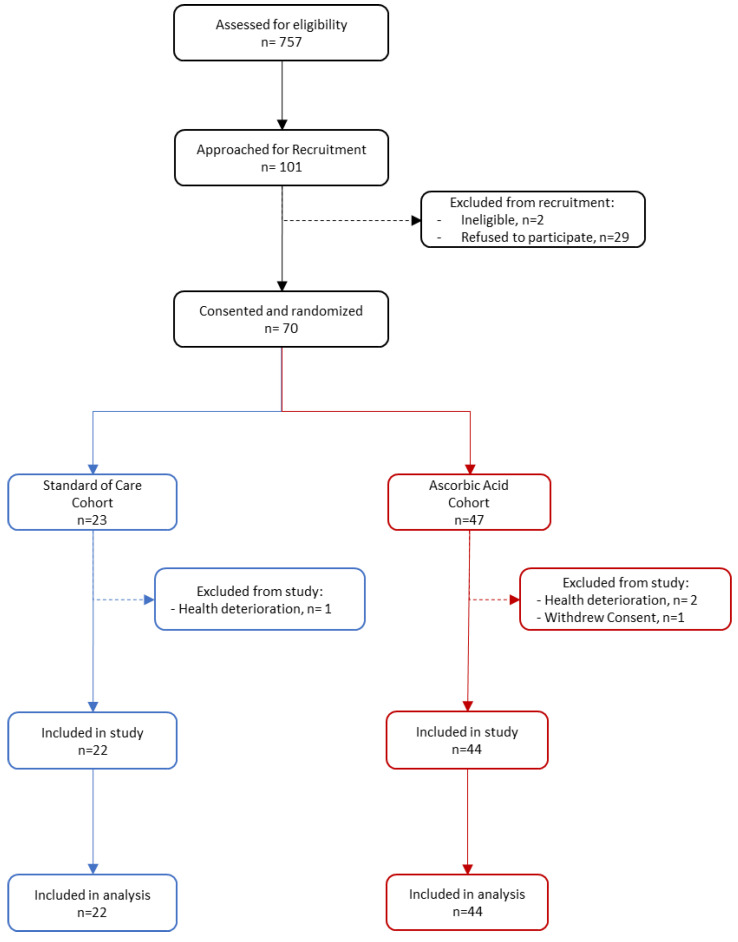
CONSORT flow diagram.

Of the 101 patients approached, seventy consented to the study and were randomized. Of those seventy, four were excluded prior to analysis (Figure 1). This included one patient withdrawal and three exclusions due to clinical deterioration between consent and randomization. Of the remaining sixty-six patients, forty-four were randomized to receive AA plus SoC and twenty-two were randomized to receive SoC alone.

In regard to demographics and baseline comorbidities, patients were generally balanced between the treatment and control arms (Table 1). The mean age in the treatment arm was 60 years compared to 61 years in the control arm. Fifty percent of the population was female in both groups. At the time of randomization, the mean body mass index was 33.5 in the treatment arm and 35 in the SoC arm.

The mean time from COVID-19-related symptom onset to randomization was 8.1 days (standard deviation 3.6) in the treatment arm and 7.2 days (standard deviation 3.9) in the SoC arm. The baseline oxygen requirement was higher in the SoC arm compared to the treatment arm (3 L/min vs. 2.5 L/min, respectively). During the peri-randomization period, six patients were febrile in the SoC arm, while only one patient was febrile in the treatment arm. Although baseline clinical characteristics were defined by randomization, the time from admission to randomization was noted to be longer in the AA group—1.9 days vs. 1.3 days, respectively. Though there were notable differences in antibiotic and non-steroidal anti-inflammatory, the current SoC for COVID-19, which includes remdesivir and corticosteroids, was balanced between the two study arms.

### 3.2. Interim Analyses

Among the first 21 patients in the AA group, only 2 (9.5%) needed high-flow oxygen and ICU-level care (upper 95% confidence limit: 27.1%). Therefore, the trial continued to its full accrual.

### 3.3. Primary Outcome

The study’s primary outcome was clinical improvement within 72 h of randomization, based on three components: 50% reduction in oxygen flow rate, 50% reduction in bronchodilator use, or discharge. The results are summarized in Table 2. The point estimate for overall clinical improvement, as defined by the primary outcome, was larger in the AA arm compared to the SoC arm (86% vs. 73%; odds ratio, OR = 2.36; 90% confidence interval, CI: 0.66 to 8.07), though the difference was not statistically significant (1-sided *p* = 0.158). When adjusted for admission-to-randomization time, the odds ratio for clinical improvement with treatment was 2.00 (90% CI, 0.57 to 7.00; 1-sided *p* = 0.219). Furthermore, time of initiation of the AA treatment did not substantially impact the results; clinical improvement within 72 h was noted in 19 of the 23 (83%) treatment-arm patients who received their first infusion within the first 3 h vs. 19 of the 21 (90%) who received their first infusion later.

Regarding the composite endpoint’s components, a 50% decrease in supplemental oxygen flow rate was observed in 77.3% of individuals in the AA arm compared to 68.2% of those in the SoC arm. Less than a third of the patients in both groups were using bronchodilators; among those who were, a 50% reduction in use was noted in 6.8% of the AA arm vs. 4.5% of the SoC arm. Finally, discharge within 72 h of randomization was 29.5% in the AA arm vs. 9.1% in the SoC arm.

### 3.4. Secondary Outcomes

Table 3 summarizes the results for the secondary endpoints of fever and adverse events. Fever was less common in the AA group compared to the SoC group (14% vs. 36%; *p* = 0.038). However, more patients in the SoC group than in the AA group had fever at enrollment; when those patients were excluded, the difference between the groups was attenuated (14% vs. 25%; *p* = 0.263). Adverse event rates were higher in the AA arm than in the SoC arm, but the difference was not significant (any adverse event: 55% vs. 32%; *p* = 0.133; serious adverse events: 18% vs. 9%; *p* = 0.293). The serious adverse events included death (one SoC), hypothermia (one AA), hypoxia (six AA, two SoC), leukocytosis (one AA), pulmonary edema (two AA), and sepsis (two AA). There were no reported study-related deaths in either arm.

**Figure 2:**
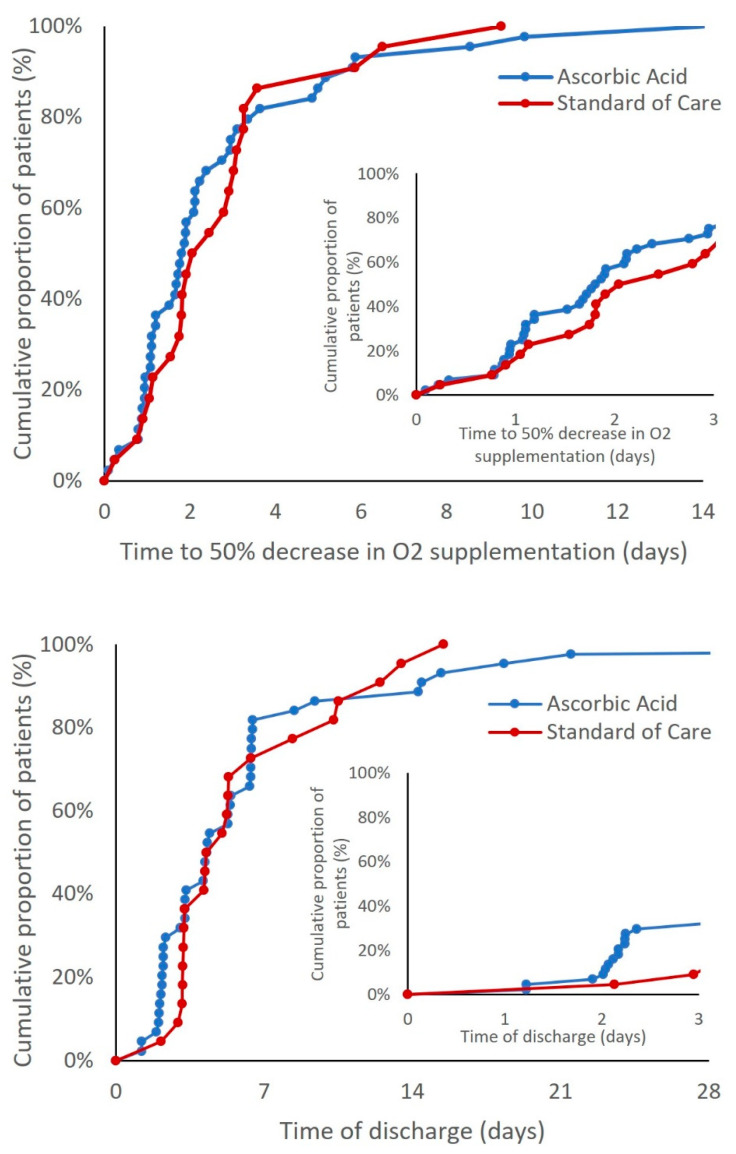
Time to a 50% reduction in oxygen supplementation and time to discharge in the study arms.

For the overall study period, the time to a 50% reduction in supplemental oxygenation was not statistically significant between the AA and SoC arms (median = 1.9 days vs. 2.2 days; hazard ratio (HR) 1.13 [90% CI, 0.65 to 1.97]; *p* = 0.334) (Figure 2). The time to discharge was also not statistically different between the two groups (median = 4.3 days vs. 4.6 days; HR 1.03 [CI 90%, 0.61 to 1.76]; *p* = 0.453) (Figure 2). Adjustment for the admission-to-randomization time did not materially change these results; the estimated hazard ratios were 1.05 (90% CI, 0.60 to 1.85; 1-sided *p* = 0.432) for the time to a 50% reduction in oxygen supplementation and 1.03 (90% CI: 0.61 to 1.76; 1-sided *p* = 0.453) for the time to discharge. Differences in the cumulative proportion curves for a reduction in supplemental oxygen and discharge were more pronounced in the first 72 h of the study (Figure 2, insets). Regarding protocol deviations, as noted in Figure 1, three patients (1 AA, 2 SoC) were incorrectly excluded from the study shortly after consent and randomization due to clinical deterioration. Notes to file were included in trial documentation.

## 4. Discussion

In this unblinded, randomized exploratory trial of hospitalized adults with COVID-19, intravenous AA plus SoC vs. SoC alone was provided to individuals admitted to a general medical ward with positive COVID-19 tests who required supplemental oxygen. Based on our primary outcomes, there was no statistical difference between those patients who received AA compared to those who received SoC for the primary outcome of clinical improvement within 72 h of randomization.

The scientific premise of this trial is that AA administered intravenously to produce pharmacologic concentrations might be an effective treatment agent if given early in the clinical course of COVID-19 and at sufficient doses. As has been previously described, AA may play a role in lymphocyte activation and proliferation [7,8,10]. This physiologic observation may be harnessed and augmented with pharmacologic AA concentrations. When given intravenously, pharmacologic AA concentrations promote the steady-state formation of hydrogen peroxide in vivo over hours [14,15]. Hydrogen peroxide, in turn, may serve as an intracellular second messenger in the antiviral activity of lymphocytes [12]. Independent of lymphocyte activation, pharmacologic AA also has direct inhibitory effects on the replication of some viruses in vitro, presumably via hydrogen peroxide formation [9]. Together, these properties of pharmacologic AA could be harnessed in the early treatment of COVID-19 pneumonia.

Several trials of AA in COVID-19 patients are in progress or under analysis [18,19,20], in addition to a clinical trial of oral AA and zinc in patients with COVID-19 that was stopped early due to futility [21]. The trial presented here is unique in that it not only examined intravenous as opposed to oral AA but is also based on the hypothesis-driven mechanism where pharmacologic ascorbic acid produces hydrogen peroxide in the extracellular fluid. The data supporting this mechanism of action and clinical dosing application are primarily from the use of pharmacologic ascorbic acid in cancer treatment. Via hydrogen peroxide formation, pharmacologic ascorbic acid concentrations selectively target cancer cell death without harm to normal tissue [14,15,16,22,23,24,25]. The use of the intravenous route was essential to our study design as oral AA does not produce pharmacologic plasma concentrations of the vitamin nor does it increase baseline hydrogen peroxide concentrations in extracellular fluid [13,23]. The dose selected was based on its ability to safely generate maximal hydrogen peroxide concentrations in vivo in extracellular fluid, utilizing pre-clinical data and data obtained from patients with malignancies who received intravenous AA treatment.

Intravenous AA has been studied as a sepsis or acute respiratory distress syndrome (ARDS) therapeutic agent in clinical trials with placebo control and randomization [26,27,28,29,30,31,32,33,34]. AA was used either as a single agent or in combination with hydrocortisone and/or thiamine. The majority of evidence from these and other studies does not show benefits from supraphysiologic AA concentrations achieved from the doses used [35,36]. In sepsis studies, the doses used generally were approximately 25 mg/kg every 6 h, much less than the dose used here of 0.9 g/kg (900 mg/kg) once daily. Similarly, in sepsis studies, estimated peak plasma concentrations are predicted to be less than 5% of the peak concentrations predicted from the dose in our COVID trial [15,23,37]. Doses used in sepsis studies were initially selected to rapidly reverse AA deficiency unrelated to the generation of hydrogen peroxide, although the use of even these comparatively lower AA doses in a sepsis population might confer increased risk because of concurrent acute kidney injury in sepsis [38,39].

Based on our hypotheses, we predicted that the benefit of AA would be most apparent in the early study period. For this reason, we constructed a primary endpoint of clinical improvement within 72 h of randomization. The endpoint included three components—a reduction in supplemental oxygen, a reduction in bronchodilator use, and discharge. At this stage in our understanding of COVID-19, a reduction in supplemental oxygen use and discharge remain viable measures of a patient’s clinical course. The Center for Disease Control and Prevention continues to monitor COVID-19-related ventilatory and discharge patterns as a component of its National Hospital Care Survey (NHCS) [40]. Furthermore, in its guideline “COVID-19: Developing Drugs and Biological Products for Treatment and Prevention”, the United States Food and Drug Administration (FDA) recommends the need for hospitalization and the reduction in supplemental oxygen as important clinical outcomes to measure in pharmaceutical trials [41]. However, the reduction of bronchodilator use is not featured in either NHCS data or in the FDA trial recommendations. This likely reflects our current understanding of the pathophysiology of COVID-19. Bronchodilator use does not appear to improve clinical outcomes in COVID-19, possibly due to its predilection for parenchymal and vascular pathology as opposed to airway disease [42].

There was no statistically significant difference between AA and SoC for the primary endpoint of early clinical improvement. Despite the lack of composite statistical significance, point estimates of the endpoint’s individual components were all supportive of the treatment arm. As shown in analyses of the secondary endpoints (Table 3, Figure 2), there were early splits in the cumulative proportion curves for the time to a 50% reduction in oxygenation and the time to discharge. These early potential benefits disappeared over time. Although these are post-hoc analyses, they hint at a possible advantage to the outcomes during the 72-h window after randomization, consistent with our hypotheses. Further consistent with our hypotheses, these trends were nullified as the curves crossed later in the hospitalization. As secondary endpoints, both the times to discharge and a 50% reduction in oxygenation were statistically non-significant when measured over the entire hospitalization. This pattern was not wholly unexpected. As noted above, an early benefit was consistent with our proposed mechanism of action.

Regarding safety, the proportion of patients who experienced a clinical decline in the treatment arm did not meet stopping rules and the trial was continued to its full accrual. Though the point estimates were higher for serious adverse events and clinical decline in the first 36 h, the study was not powered to detect these differences, which were not statistically significant.

Our trial had limitations. In retrospect, the study was small with optimistic outcome assumptions. Outcomes in this trial were defined in the early part of the pandemic, and do not reflect our current understanding of COVID-19 as a disease process. Furthermore, our patient risk stratification, while focused on age and comorbidities, may be improved in future studies. An emphasis on more severe underlying metabolic disease (i.e., diabetes), as opposed to any pulmonary or cardiovascular disease, may better improve patient selection and risk analyses. Viral load and specific analyses of lymphocyte populations were not feasible for practical and funding limitations, and these data may guide potential future trials of pharmacologic AA. As has become important both in clinical trials and in clinical practice, more consideration for symptom onset and baseline inflammatory markers, such as D-dimer and C-reactive protein, may lead to better correlates for clinical practice. Such considerations can strengthen future study designs for AA in the treatment of COVID-19.

Although the primary endpoint was not met in this study, trends in supplemental oxygen use and patient discharge suggest possible clinical signals consistent with the action of AA at pharmacologic doses. Even at these high doses, there was no statistical difference in safety between the two study arms. There is a continuing need for early treatment of COVID-19 in hospitalized patients, and this need will only increase as viral variants evolve. We believe that future larger studies exploring the use of pharmacologic AA are warranted and should account for its early disease effects and our evolving understanding of COVID-19 infection.

## Figures and Tables

**Table 1:** Demographic and clinical characteristics of patients in the treatment and control groups.

	Number (%)	
	Standard of Care Alone (*N* = 22)	Ascorbic Acid Plus Standard of Care (*N* = 44)
Characteristic		
Mean age (SD), years	61 (11)	60 (17)
Age groups, years		
<50	3 (13.6)	13 (29.5)
50–59	5 (22.7)	7 (15.9)
60–69	10 (45.5)	13 (29.5)
70+	4 (18.2)	11 (25.0)
Sex		
Female	11 (50.0)	22 (50.0)
Male	11 (50.0)	22 (50.0)
Race/Ethnicity		
Caucasian/White, Non-Hispanic	14 (63.6)	23 (52.3)
African American/Black, Non-Hispanic	4 (18.2)	17 (38.6)
Asian, Non-Hispanic	1 (4.5)	2 (4.5)
Hispanic	3 (13.6)	2 (4.5)
Mean BMI (SD), kg/m^2^	35.0 (8.7)	33.5 (8.8)
BMI groups, kg/m^2^		
Normal weight (18.5–24.9)	2 (9.1)	6 (13.6)
Overweight (25–29.9)	5 (22.7)	11 (25.0)
Obese (30+)	15 (68.2)	27 (61.4)
Smoking status		
Never smoker	12 (57.1)	23 (52.3)
Former smoker	9 (42.9)	17 (38.6)
Current smoker	0 (0.0)	4 (9.1)
Comorbid conditions		
Diabetes	8 (36.4)	15 (34.9)
Cardiovascular disease	12 (54.5)	29 (65.9)
Chronic obstructive pulmonary disease	5 (22.7)	8 (18.6)
Organ transplant recipient	1 (4.5)	0 (0.0)
Risk of complications		
Low	4 (18.2)	9 (20.5)
High	18 (81.8)	35 (79.5)
Mean time since the onset of symptoms (SD), days	7.2 (3.9)	8.1 (3.6)
Medications administered after randomization		
Antibiotics	2 (9.0)	9 (20.5)
Remdesivir	20 (90.9)	41 (93.2)
Glucocorticoids	18 (81.8)	33 (75.0)
NSAIDS	11 (50.0)	8 (18.1)

BMI, body mass index; kg/m^2^, kilogram per meter squared; NSAIDS, non-steroidal anti-inflammatory drugs; SD, standard deviation.

**Table 2:** Primary endpoint.

	Standard of Care Alone (*N* = 22)	Ascorbic Acid Plus Standard of Care (*N* = 44)	Odds Ratio (90% Confidence Interval)	*p*-Value
Number of patients who achieved clinical improvement within 72 h of randomization (*N*)	16 (72.7%)	38 (86.4%)	2.36 (0.66, 8.07)	0.158
Number of patients who achieved a 50% reduction in supplemental oxygen (*N*)	15 (68.2%)	34 (77.3%)		
Number of patients who achieved a 50% reduction in bronchodilator use (*N*)	1 (4.5%)	3 (6.8%)		
Number of patients discharged (*N*)	2 (9.1%)	13 (29.5%)		

**Table 3:** Secondary endpoints.

	Standard of Care Alone (*N* = 22)	Ascorbic Acid Plus Standard of Care (*N* = 44)	Hazard Ratio (90% Confidence Interval)	*p*-Value
Time to 50% reduction in supplemental oxygen (days)	2.24	1.87	1.13 (0.65, 1.97)	0.334
Time to discharge (days)	4.65	4.3	1.03 (0.61, 1.76)	0.453
Number of patients with any fever (*N*)	8 (36.4%)	6 (13.6%)	0.28 (0.09, 0.93)	0.038
Number of patients with any fever, excluding patients with fever at randomization (*N*)	4 (25.0%)	6 (14.0%)	0.50 (0.13, 2.15)	0.263
Any serious adverse event (*N*)	2 (9.1%)	8 (18.2%)	2.23 (0.46, 15.5)	0.293
Clinical decline within 36 h of randomization (*N*)	2 (9.1%)	4 (9.1%)	0.95 (0.16, 7.84)	0.643

## Data Availability

The data presented in this study are available on request from the corresponding author. The data are not publicly available due to our institution’s patient privacy policies.

## Supplementary Materials

The following supporting information can be downloaded at: https://www.mdpi.com/2075-1729/12/3/453/s1, Table S1: CONSORT 2010 checklist of information to include when reporting a randomised trial.

## Abbreviations

AA	ascorbic acid, vitamin C
COVID-19	coronavirus disease 2019
SARS-CoV-2	severe acute respiratory syndrome coronavirus 2
SoC	standard of care
