# Correction: Coppock et al. Pharmacologic Ascorbic Acid as Early Therapy for Hospitalized Patients with COVID-19: A Randomized Clinical Trial. *Life* 2022, *12*, 453

**DOI:** 10.3390/life12091354

**Published:** 2022-08-31

**Authors:** Dagan Coppock, Pierre-Christian Violet, Gustavo Vasquez, Katherine Belden, Michael Foster, Bret Mullin, Devon Magee, Isabelle Mikell, Lokesh Shah, Victoria Powers, Brian Curcio, Daniel Monti, Mark Levine

**Affiliations:** 1 Division of Infectious Diseases, Department of Medicine, Sidney Kimmel Medical College, Thomas Jefferson University, 1015 Chestnut Street, Philadelphia, PA 19107, USA; 2 Molecular and Clinical Nutrition Section, National Institute of Diabetes and Digestive and Kidney Diseases, National Institutes of Health, 10 Center Drive, Bethesda, MD 20892, USA; 3 Jefferson Clinical Research Institute, Thomas Jefferson University, 833 Chestnut Street, Philadelphia, PA 19107, USA; 4 Sidney Kimmel Medical College, Thomas Jefferson University, 1015 Walnut Street, Philadelphia, PA 19107, USA; 5 Division of Biostatistics, Department of Pharmacology and Experimental Therapeutics, Sidney Kimmel Medical College, Thomas Jefferson University, 1015 Chestnut Street, Philadelphia, PA 19107, USA; 6 Department of Integrative Medicine and Nutritional Sciences, Sidney Kimmel Medical College, Thomas Jefferson University, 925 Chestnut Street, Philadelphia, PA 19107, USA

## Deletion of an Author

The authors wish to make a change to the author names (deleting one author—Constantine Daskalakis) for this paper [1]. Daskalakis has requested to be removed from the article. The correct authorship is as below:

Dagan Coppock ^1,^*, Pierre-Christian Violet ^2^, Gustavo Vasquez ^1^, Katherine Belden ^1^, Michael Foster ^3^, Bret Mullin ^3^, Devon Magee ^3^, Isabelle Mikell ^4^, Lokesh Shah ^4^, Victoria Powers ^4^, Brian Curcio ^5^, Daniel Monti ^6^ and Mark Levine ^2,^*

The “Author Contributions” statement thus should be updated to the following version:

**Author Contributions:** P.-C.V., D.M. (Daniel Monti) and M.L. conceived the study; D.C., D.M. (Daniel Monti), M.L. and M.F. developed the trial methodology; D.C., G.V., K.B. and B.M. reviewed and enrolled subjects; D.C., D.M. (Daniel Monti), G.V. and K.B. provided clinical oversight and monitored safety; B.M., D.M. (Devon Magee), I.M., L.S. and V.P. were responsible for preparing and curating data; B.C. and D.C. performed formal analyses; D.C. and M.L. prepared the original manuscript draft; all authors reviewed and edited the manuscript; D.M. (Daniel Monti) acquired funding; D.C., D.M. (Daniel Monti) and M.L. supervised overall efforts. All authors have read and agreed to the published version of the manuscript.

## Text Correction

The second correction was the inclusion of the words “study-related” to the sentence: “There were no reported deaths in either arm.” This sentence has been changed to now read: “There were no reported study-related deaths in either arm.”

The authors apologize for any inconvenience caused and state that the scientific conclusions are unaffected. This correction was approved by the Academic Editor. The original publication has also been updated.
